# Atypical *Brucella inopinata*–Like Species in 2 Marine Toads

**DOI:** 10.3201/eid2706.204001

**Published:** 2021-06

**Authors:** Raisa A. Glabman, Kimberly A. Thompson, Rinosh Mani, Ryan Colburn, Dalen W. Agnew

**Affiliations:** National Institutes of Health, Bethesda, Maryland, USA (R.A. Glabman);; Michigan State University, East Lansing, Michigan, USA (R.A. Glabman, K.A. Thompson, R. Mani, R. Colburn, D.W. Agnew;; Binder Park Zoo, Battle Creek, Michigan, USA (K.A. Thompson);; John Ball Zoo, Grand Rapids, Michigan, USA (R. Colburn)

**Keywords:** *Brucella*, brucellosis, oophoritis, amphibian, toad, zoonoses, bacteria, United States

## Abstract

We describe the isolation of atypical *Brucella inopinata*–like species and unique clinicopathologic findings in 2 adult marine toads (*Rhinella marina*), including oophoritis in 1 toad. These findings represent a novel emerging disease in toads and a possible zoonotic pathogen.

Brucellosis is a worldwide zoonosis caused by gram-negative, intracellular *Brucella* coccobacilli. Expanding from 6 species classically associated with abortion in mammals (*B. melitensis*, *B. suis*, *B. abortus*, *B. ovis*, *B. canis*, and *B. neotomae*), the genus now includes novel strains from marine mammals (*B. ceti*, *B. pinnipedialis*), baboons (*B. papionis*), and foxes (*B. vulpis*). Two of these (*B. ceti*, *B. pinnipedialis*) are also considered atypical *Brucella* species similar to *B. microti* and *B.* *inopinata* (*1*). Atypical *Brucella* lesions in humans, wild mammals, amphibians, and fish range from localized manifestations to systemic infection with high death rates (*2**–**8*); however, reproductive lesions more typical of mammalian brucellosis are rare in amphibians. Previous reports of *Brucella* in amphibians have also included asymptomatic infections, suggesting that *Brucella* may be a commensal microorganism or opportunistic pathogen (*9*). The precise epidemiology, pathogenesis, and zoonotic potential of *Brucella* in amphibians remains largely unknown. We report atypical *Brucella* infection in 2 marine toads.

Cases 1 and 2 originated from the same captive breeding marine toad (*Rhinella marina*) colony; the 2 toads cohabited before toad 1’s transfer to a different zoological institution, resulting in a 4-year period with no contact before death. Case 1 was in an adult female marine toad with a 1.5-cm subcutaneous mass near the parotid gland. A second mass was palpated within the coelom, and ultrasound suggested ovarian origin. The toad was anesthetized for exploratory celiotomy, and both masses were excised and submitted for histopathology and culture. The coelomic mass was encapsulated within the left ovary, measured 3 × 2 × 2 cm, and contained purulent material (Figure). Histologically, the masses contained multifocal regions of necrosis and amorphous eosinophilic material with sheets of macrophages containing numerous intracytoplasmic, gram-negative, non–acid-fast coccobacilli (Appendix Figure). Diagnosis led to euthanasia; postmortem findings included mild coelomic effusion, lymphohistiocytic pericarditis, and fibrinous peritonitis. Case 2 was in an adult male marine toad, which was submitted for necropsy after being found dead in its enclosure. The toad was in poor body condition with no other lesions found on gross and histologic examination.

**Figure:**
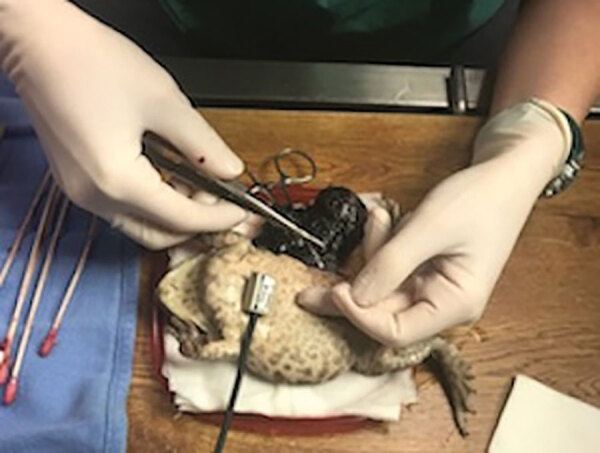
Celiotomy to remove ovarian mass in a marine toad (case 1). The mass was later diagnosed as *Brucella inopinata*–like oophoritis.

We used fresh ovarian tissue from the female toad and pooled fresh tissues (liver, kidney, and spleen) from the male toad for bacterial culture. We incubated duplicate culture plates at 37°C with 5% CO_2_ and in ambient conditions (21 + 2°C, no CO_2_). After 24 hours of ovarian mass culture incubation, we observed numerous pure bacterial colonies on blood and MacConkey agar at both 37°C and ambient conditions (colony size was smaller at ambient temperature). The pooled samples from the male toad contained mixed bacterial cultures after 24 hours of incubation. We identified the bacterial colony from the female toad with matrix-assisted laser desorption ionization-time of flight mass spectrometry (Microflex LT; Bruker Daltonics, https://www.bruker.com) as *Brucella* sp. (isolate no. 3278), whereas we identified bacterial colonies from the male toad as *Brucella* sp. (isolate no. 5043). Both *Brucella* isolates grew on MacConkey, Thayer-Martin, and blood agar at both 37°C and ambient conditions. The isolates were positive for catalase, oxidase, urease (<5 min), and hydrogen sulfide production and negative on gel formation test (Appendix). We used the DNA from isolates 3278 and 5043 for the *Brucella* Laboratory Response Network real-time PCR; the DNA tested positive for all 3 targets. We performed partial 16S rDNA PCR assays on isolates 3278 and 5048 and partial recA PCR on isolate 3278. For both isolates, the 16s rDNA sequences had 100% sequence similarity to an atypical *Brucella* sp. isolated from a big-eyed tree frog in Germany (GenBank accession no. HE608873) (*5*). The sequences from both isolates had only 95% coverage and 99.8% sequence similarity to *B. inopinata* strain BO1 (GenBank accession no. NR116161) (*10*). The recA sequence of isolate 3278 had 100% sequence similarity to the *Brucella* sp. isolated from a big-eyed tree frog (GenBank accession no. HE608874). The recA sequence had only 64% coverage and 98.71% sequence similarity to *B. inopinata* strain BO1 (GenBank accession no. FM177719). We submitted the DNA sequences from isolates 3278 and 5043 to GenBank (accession nos. MT471347, MT471348, and MT482342).

We isolated atypical *Brucella inopinata*–like sp. from 2 adult marine toads, one an asymptomatic carrier and the second with oophoritis, a classic lesion described in mammalian *Brucella* infections. Our results suggest that marine toads are another amphibian species susceptible to atypical *Brucella* bacteria and that infection can result in long-term asymptomatic carriers as well as more typical reproductive lesions. Furthermore, this organism was isolated in 2 toads from different zoological institutions but with identical origin, suggesting that infection originated from a common source at least 4 years previously. After leaving the breeding colony, all toads were housed only with conspecifics and, for a short period of time, with one other species group. Skin swab specimens from all other contacted amphibians at the zoos tested negative for *Brucella*. Diet consisted of a variety of insect species, making 2 separate introductions of *Brucella* from an outside source possible but unlikely. These findings highlight the need for additional testing of atypical *Brucella* spp., a potential emerging disease in amphibians, and warrants precautions when handling amphibians because of the potential for zoonoses.

Appendix Detailed methods for molecular and biochemical characterization of atypical *Brucella inopinata*–like species.
