# Supplementary material for: Atypical Brucella inopinata–Like Species in 2 Marine Toads
Source: Emerg Infect Dis. 2021 Jun;27(6):1748–50. doi: 10.3201/eid2706.204001 (PMC8153883; doi:10.3201/eid2706.204001)
Supplement: Appendix — Detailed methods for molecular and biochemical characterization of atypical Brucella inopinata–like species. [file 20-4001-Techapp-s1.pdf]

Article DOI: https://doi.org/10.3201/eid2706.204001

# Atypical *Brucella inopinata*–Like Species in 2 Marine Toads

## Appendix

## Methods

We used a Qiagen DNeasy tissue kit (Qiagen, https://www.qiagen.com) to extract DNA from suspect *Brucella* sp. We used the extracted DNA for a Laboratory Response Network real-time assay and partial 16S rDNA and recA PCR using previously published primers (*1*,*2*). We treated PCR products with ExoSAP-IT Express (Thermo Fisher, https://www.thermofisher.com) and used both forward and reverse primers for sequencing at Eurofins Genomics (Eurofins Genomics, https://www.eurofins.com). We trimmed the resulting sequences and analyzed the consensus sequence with NCBI BLAST (http://blast.ncbi.nlm.nih.gov/Blast.cgi) against the nucleotide database. The biochemical characterization of the bacteria included lead acetate, oxidase, catalase (Remel, http://www.remel.com), and gel formation tests (gel formation of the bacterial isolate suspension in 1% phenolized saline) (*3*).

### References

1. Scholz HC, Nöckler K, Göllner C, Bahn P, Vergnaud G, Tomaso H, et al. *Brucella inopinata* sp. nov., isolated from a breast implant infection. Int J Syst Evol Microbiol. 2010;60:801–8. PubMed https://doi.org/10.1099/ijs.0.011148-0

2. Kiupel M, Desjardins DR, Lim A, Bolin C, Johnson-Delaney CA, Resau JH, et al. Mycoplasmosis in ferrets. Emerg Infect Dis. 2012;18:1763–70. PubMed https://doi.org/10.3201/eid1811.120072

3. Lawaczeck E, Toporek J, Cwikla J, Mathison BA. *Brucella canis* in a HIV-infected patient. Zoonoses Public Health. 2011;58:150–2. PubMed https://doi.org/10.1111/j.1863-2378.2010.01334.x

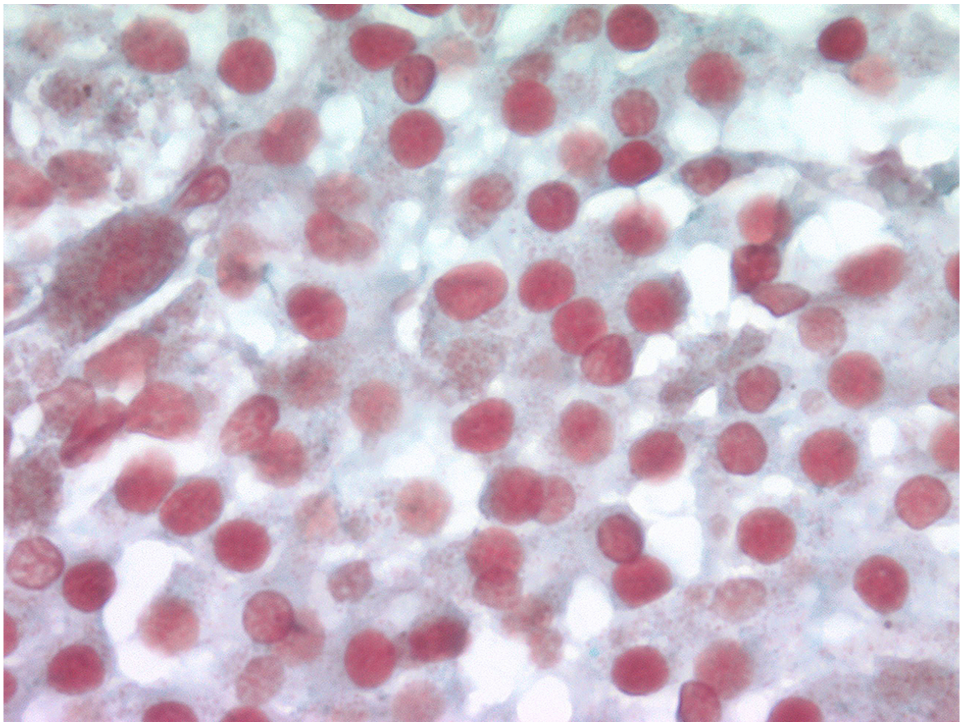

**Appendix Figure.** Case 1: Gram stain of numerous intracytoplasmic *Brucella* sp. coccobacilli within macrophages, which were also acid-fast negative (not shown).
